# Chin Necrosis as a Consequence of Prone Positioning in the Intensive Care Unit

**DOI:** 10.1155/2015/762956

**Published:** 2015-02-24

**Authors:** Daniel Lee John Bunker, Michael Thomson

**Affiliations:** ^1^Department of Plastic Surgery, Launceston General Hospital, 274-280 Charles Street, Launceston, TAS 7250, Australia; ^2^Department of Plastic Surgery, St. Vincent's Hospital, 390 Victoria Street, Darlinghurst, Sydney, NSW 2010, Australia; ^3^School of Medicine, University of Tasmania, Launceston, TAS 7250, Australia

## Abstract

Pressure necrosis of the skin is a rarely reported avoidable complication of prone positioning that can be minimised by active collaboration between care teams. We report a case of pressure necrosis of the chin after prone ventilation in the intensive care setting. Such injuries pose a risk of infection, possible need for surgical intervention, and increased costs to the health care system. Pressure necrosis injuries should be diligently guarded against by the careful selection of support devices, frequent turning, and rigorous skin care to minimise extended external compression, particularly on the face and bony prominences.

## 1. Introduction

Prone positioning can be used in the intensive care setting to improve ventilation or during anaesthesia to optimise access to the surgical site. Pressure injuries are a well-recognised and a theoretically preventable complication of prone positioning which in their most severe form can culminate in pressure necrosis. Aetiological factors include the duration and amount of pressure, friction or shearing forces, and tissue perfusion pressure [1]. Such injuries can act as a source of sepsis, requiring protracted hospital stay or surgical intervention, ultimately leading to increased morbidity for the patient and increased costs of care. We present a patient who developed pressure necrosis of the chin after prone ventilation for severe viral pneumonia. The presentation and management are discussed, along with suggestions for prevention.

## 2. Case Presentation

A 73-year-old man was admitted with a three-week history of worsening shortness of breath on exertion, rhinorrhoea, and a cough productive of white sputum. He reported 4 days of chills and rigors at home not improving on outpatient oral antibiotic therapy. He reported recent travel through Europe over the preceding few months and was an ex-smoker. On arrival to the hospital emergency department, he was markedly hypoxic and tachypnoeic, with a leukocytosis and acute renal impairment. Chest X-ray revealed bilateral infiltrates that were worse on the right. Given his marked hypoxia, he was admitted to the intensive care unit with a diagnosis of severe viral pneumonia and type 1 respiratory failure. Empirical treatment with multiple intravenous antibiotics was commenced immediately for chest sepsis; however, he developed worsening respiratory failure and was intubated on day 4 of admission. Despite prostacyclin nebulisers and nitric oxide, he remained having difficulty to oxygenate and the decision was made to turn the patient prone, with the bed tilted head up to aid the pressure care. The face was supported in a gel ring. He was kept prone for a total of 36 hours, with a 12 hourly turning regime.

**Figure 1:**
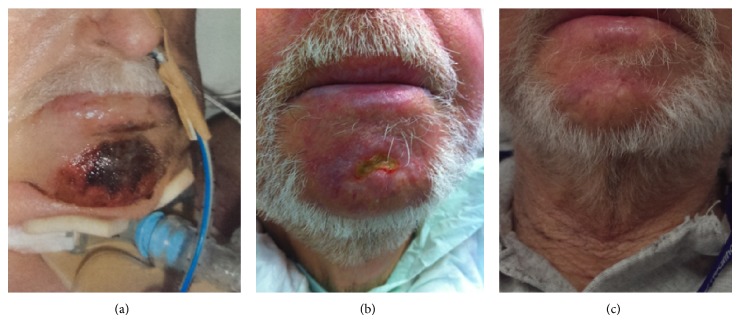
Pressure necrosis of the chin, noted first as a bruise (day 6), which developed an eschar which was left to autodebride (2 months) and later healed leaving a scar (3 months).

Pressure areas were subsequently noted across his chin (5 × 4 centimeters, unable to determine depth) and forehead (9 × 1 centimeters, grade II). The wound care nurse and plastics team were consulted, and the pressure areas were monitored and treated with paraffin and chloramphenicol ointment for the next 12 days. The forehead wound healed; however, the pressure area on the chin became necrotic. The necrotic area subsequently formed a dark eschar. The decision was made to leave the area to autodebride and heal from the periphery, which occurred slowly over the ensuring 3 months to leave an area of scar with patchy alopecia (see Figure 1). Such scarring on the face can present cosmetic issues due to loss of hair follicles in the man or unsightly scar tissue in the female.

## 3. Discussion

The prone position provides exposure advantages for surgery of the spine or posterior chest wall and lower limbs and can also be employed to assist with ventilation, particularly in patients with acute respiratory distress syndrome. Its use provides particular challenges from a practical point of view due to difficulties in access to the airway, monitoring, and catheters. Physiological changes in haemodynamics and ventilation are frequently observed in prone patients. Cardiac output is reduced as a result of decreased venous return, reduced ventricular compliance, and increased intrathoracic pressure [2]. Furthermore, prone positioning reduces disparity in ventral and dorsal pleural pressures, minimising alveolar overinflation and collapse and possibly improving functional residual capacity, leading to improved ventilation and increased arterial oxygen saturations [3–5].

Complications of prone positioning include neurovascular injuries, injuries to the eyes and ears, and pressure necrosis of the skin [2, 4, 6–8]. Pressure necrosis occurs from long-term exclusion of blood flow to tissues as a result of external compression. It therefore affects areas of dependent contact, necessitating careful attention to the face, chest wall, genitalia, and bony prominences such as the iliac crests. Whilst pressure necrosis is well-recognised as an avoidable complication of prone positioning, case reports of pressure injuries are relatively rare [2]. Reported injuries to the face usually involve the malar regions, lips (possibly augmented by endotracheal tube placement), nose, forehead, and tongue [7, 9–11].

While it has been long recognised as a potential complication, this is the first care report of chin necrosis as a consequence of prone positioning to the authors' knowledge. Heightened awareness needs to be maintained to prevent pressure areas from developing. Rolling the patient intermittently relieves periods of extended pressure, although this is not always feasible from a practical point of view (during surgery) and does not guarantee avoidance of complications as exemplified by this case. Patients within the intensive care unit are at particular risk of developing pressure injuries due to protracted immobilisation, reduced tissue perfusion pressures, systemic illness, reduced physiological reserves, and impaired healing ability. As such, prevention of such injuries should be an active part of conscious care, with active identification of patients at increased risk, implementation of suitable preventative measures, and close examination of these patients as a part of their daily care [1].

The use of the Concorde position and a cervical collar have been attributed as risk factors for developing pressure necrosis of the chin in the prone position [12]. A study of facial tissue pressures in the prone position revealed that pressure effects can be reduced by careful selection of an appropriate head rest, with a polyurethane foam head rest within a protected helmet system advocated [13]. Möllmann and colleagueshave advocated for a transparent operating table with a foam-cushion face mask to facilitate intraoperative monitoring [14].

Conscious collaborations between the care team regarding the necessity and length of prone positioning are vital steps to minimise these iatrogenic injuries. We suggest that preventative measures for developing pressure necrosis in the prone position begins with active identification of high risk patients and careful selection of appropriate support devices on a case-dependent basis, with regular monitoring for signs of pressure injuries incorporating frequent turning. Maintenance of tissue perfusion pressures and attention to body temperature are also important to avoid compounding the effects of pressure towards local ischaemia.

## References

[1] Reilly E. F., Karakousis G. C., Schrag S. P. (2007). Pressure ulcers in the intensive care unite: the ‘forgotten’ enemy. *Scientist*.

[2] Edgcombe H., Carter K., Yarrow S. (2008). Anaesthesia in the prone position. *British Journal of Anaesthesia*.

[3] Lai-Fook S. J., Rodarte J. R. (1991). Pleural pressure distribution and its relationship to lung volume and interstitial pressure. *Journal of Applied Physiology*.

[4] Knight D. J. W. (2004). Patient positioning in anaesthesia. Continuing education in anaesthesia. *Critical Care & Pain*.

[5] Douglas W. W., Rehder K., Beynen F. M., Sessler A. D., Marsh H. M. (1977). Improved oxygenation in patients with acute respiratory failure: the prone position. *The American Review of Respiratory Disease*.

[6] Curley M. A. Q. (1999). Prone positioning of patients with acute respiratory distress syndrome: a systematic review. *The American Journal of Critical Care*.

[7] Offner P. J., Haenel J. B., Moore E. E., Biffl W. L., Franciose R. J., Burch J. M. (2000). Complications of prone ventilation in patients with multisystem trauma with fulminant acute respiratory distress syndrome. *The Journal of Trauma*.

[8] Yu H.-D., Chou A.-H., Yang M.-W., Chang C.-J. (2010). An analysis of perioperative eye injuries after nonocular surgery. *Acta Anaesthesiologica Taiwanica*.

[9] Weis K. H. (1964). Threatening necrosis of the tip of the tongue during long-term anesthesia in prone position. *Der Anaesthesist*.

[10] Jain V., Bithal P. K., Rath G. P. (2007). Pressure sore on malar prominences by horseshoe headrest in prone position. *Anaesthesia and Intensive Care*.

[11] Alsiddiky A. (2011). Lip necrosis as a complication of a prone position in scoliosis surgery. *Sultan Qaboos University Medical Journal*.

[12] Rozet I., Vavilala M. S. (2007). Risks and benefits of patient positioning during neurosurgical care. *Anesthesiology Clinics*.

[13] Grisell M., Place H. M. (2008). Face tissue pressure in prone positioning: a comparison of three face pillows while in the prone position for spinal surgery. *Spine (Phila Pa 1976)*.

[14] Möllmann M., Henning M., Liljenqvist U., Wenk M. (2007). A foam-cushion face mask and a see-through operation table: a new set-up for face protection and increased safety in prone position. *British Journal of Anaesthesia*.

